# Estimation of Energy Expenditure in Wheelchair-Bound Spinal Cord Injured Individuals Using Inertial Measurement Units

**DOI:** 10.3389/fneur.2018.00478

**Published:** 2018-07-03

**Authors:** Werner L. Popp, Lea Richner, Michael Brogioli, Britta Wilms, Christina M. Spengler, Armin E. P. Curt, Michelle L. Starkey, Roger Gassert

**Affiliations:** ^1^Rehabilitation Engineering Laboratory, Department of Health Sciences and Technology, ETH Zurich, Zurich, Switzerland; ^2^Spinal Cord Injury Center, Balgrist University Hospital, Zurich, Switzerland; ^3^Exercise Physiology Lab, Department of Health Sciences and Technology, ETH Zurich, Zurich, Switzerland; ^4^Department of Internal Medicine I, University of Lübeck, Lübeck, Germany; ^5^Department of Clinical Neurosciences & MRC Centre for Stem Cell Biology and Regenerative Medicine, University of Cambridge, Cambridge, United Kingdom

**Keywords:** energy expenditure, spinal cord injury, inertial measurement unit, long-term activity monitoring, wheelchair, estimation model

## Abstract

A healthy lifestyle reduces the risk of cardio-vascular disease. As wheelchair-bound individuals with spinal cord injury (SCI) are challenged in their activities, promoting and coaching an active lifestyle is especially relevant. Although there are many commercial activity trackers available for the able-bodied population, including those providing feedback about energy expenditure (EE), activity trackers for the SCI population are largely lacking, or are limited to a small set of activities performed in controlled settings. The aims of the present study were to develop and validate an algorithm based on inertial measurement unit (IMU) data to continuously monitor EE in wheelchair-bound individuals with a SCI, and to establish reference activity values for a healthy lifestyle in this population. For this purpose, EE was measured in 30 subjects each wearing four IMUs during 12 different physical activities, randomly selected from a list of 24 activities of daily living. The proposed algorithm consists of three parts: resting EE estimation based on multi-linear regression, an activity classification using a k-nearest-neighbors algorithm, and EE estimation based on artificial neural networks (ANNs). The mean absolute estimation error for the ANN-based algorithm was 14.4% compared to indirect calorimeter measurements. Based on reference values from the literature and the data collected within this study, we recommend wheeling 3 km per day for a healthy lifestyle in wheelchair-bound SCI individuals. Combining the proposed algorithm with a recommendation for physical activity provides a powerful tool for the promotion of an active lifestyle in the SCI population, thereby reducing the risk for secondary diseases.

## Introduction

The life expectancy of individuals with a spinal cord injury (SCI) has increased significantly over the last decades (1). Nowadays, the leading causes of death are not a direct consequence of injury-related complications (sepsis, respiratory or renal complications) but rather related to cardiovascular disease (2, 3). Obesity, hypertension, hyperlipidemia, and diabetes have all been identified as risk factors for cardio-vascular disease, with a higher prevalence in the SCI population (4). Regular physical activity has been associated with a reduction of these risk factors in the able-bodied population, as well as in the SCI population (5–7). Unfortunately, the SCI population is challenged due to their limited choice of activities (8, 9), and there is a great need to promote and coach physical activity (10). One possible approach to promote a more active lifestyle is to provide feedback on daily physical activity and energy expenditure (EE).

Vanhees et al. showed that accelerometers and inertial measurement units (IMUs) can be used as objective assessment tools for physical activity and EE in the able-bodied population (11). Over the past 20 years, different approaches to estimate EE from IMU or accelerometer data have been proposed, ranging from simple linear regression models based on activity counts (AC) (12–14), to complex non-linear regression models based on more advanced statistical features (15), as well as approaches using artificial neural networks (ANNs) (16, 17). To increase the accuracy of EE estimation, additional sensors such as heart rate (HR) monitors to compensate for weight-loading activities (18), or air pressure sensors to improve the estimation for activities involving altitude changes were added (19). Despite the improvements in the field of EE estimation based on accelerometers and IMUs, commercially available devices show a wide range of estimation accuracy (root mean squared error of 14–28% in EE estimation) (20). However, considering that many individuals with SCI present different movement characteristics due to the use of a wheelchair, the application of methods developed in able-bodied populations will likely result in a moderate to poor EE estimation in individuals with SCI.

Although accelerometer-based EE estimation models were developed for subjects with SCI using a manual wheelchair (21, 22), combining accelerometer and demographic variables, these studies focused on a restricted set of activities and were only validated in a semi-structured environment. In a more recent study, linear regression models for the estimation of EE became available based on recordings of 20 different activities, with a mean absolute error (MAE) of around 25% (23). Using methods that are more sophisticated the quality of the EE estimation may be improved.

The main aim of this study therefore was to develop and validate an EE estimation model for wheelchair-bound SCI individuals based on non-obstructive IMU recordings in a natural setting. We included a comprehensive set of 24 different physical activities, covering a broad range of activities of daily living. Furthermore, the collected data formed a basis for a recommendation that could promote a healthy lifestyle in the wheelchair-bound SCI population.

## Methods

### Subjects

Thirty chronic SCI subjects (age 45.4 ± 11.4 years, 11 tetraplegics, 19 paraplegics) who rely on a wheelchair for daily ambulation were recruited. Inclusion criteria were an age over 18 years old and suffering from SCI for more than 6 months post injury. Subjects with all neurological levels of injury (NLI) according to the International Standard for Neurological Classification of Spinal Cord Injury (ISNCSCI), and ASIA Impairment Scale (AIS) grades (A, B, C, and D) were included (Table 1). Participants with an AIS grade D relied on a wheelchair for daily ambulation either because of hemiplegia of the lower limb or due to personal preference. Exclusion criteria were any neurological diseases other than SCI, metabolic, orthopedic or rheumatologic diseases as well as pre-morbid or ongoing psychiatric disorder. All subjects gave written informed consent in accordance with the Declaration of Helsinki prior to participating in the experiment. The study was approved by the local ethics committee of the canton of Zurich (KEK-ZH Nr. 2013-0202).

**Table 1:** Demographics and assessment scores of the included participants.

**Variables**	**Values**
No of participants	30
Age (years)	45.4 ± 11.4 (27–74)
Weight (kg)	74.3 ± 17.1 (45.6–116.8)
Height (m)	1.76 ± 0.09 (1.54–2.03)
Reported hours of sport/week	2.5 ± 2.9 (0.0–10.0)
**SEX**	
Male	27
Female	3
**INJURY LEVEL**	
C3–C8	11
T1–L1	19
**AIS SCORE: TOTAL (PARAPLEGIC/TETRAPLEGIC)**	
A	17 (14/3)
B	7 (4/3)
C	3 (1/2)
D	3 (0/3)

*Age, height, weight, and reported hours of sports/week are presented as mean ± standard deviation (range)*.

### Measurement devices

#### Activity monitor

**Figure 1:**
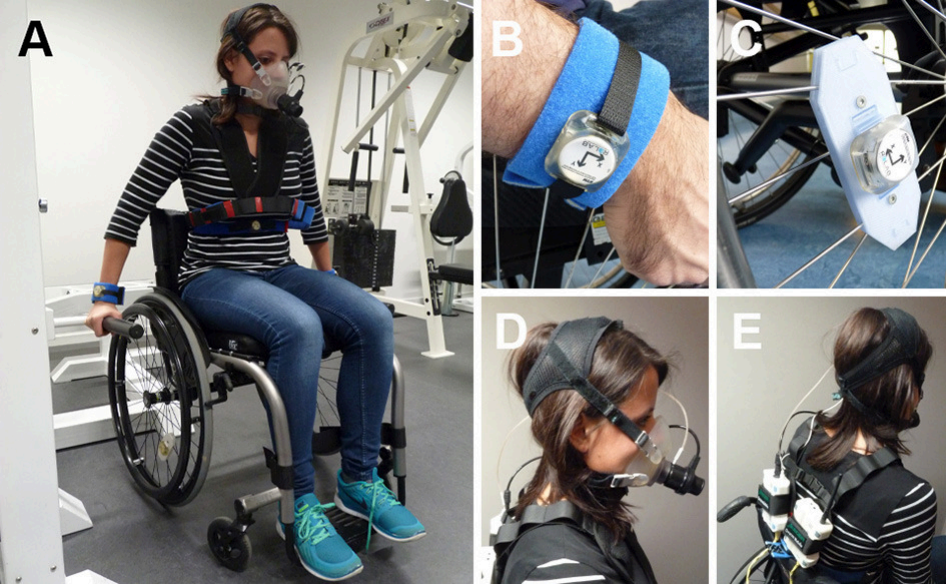
**(A)** Examiner wearing the full experimental setup during the activity weight lifting. One sensor module was attached at each wrist, one at the chest and one on the wheel of the wheelchair. **(B)** Sensor worn at the wrist with the AlphaStrap Blue and Velcro Strap fixation. **(C)** Wheel sensor with dedicated attachment. **(D)** Oxycon mobile mouth piece. **(E)** Back view of the Oxycon Mobile with sensor box and data exchange unit. Written and informed consent was obtained for the publication of these images.

An IMU (ReSense) developed by Leuenberger and Gassert was used for this study (Figures 1B,C) (24). The sensor consists of a 3-axis accelerometer, a 3-axis gyroscope, a 3-axis magnetometer (not used in this study), as well as a barometric pressure sensor for altitude estimation. This low-power 10-degrees-of-freedom IMU can continuously record data for around 48 h at a sampling rate of 50 Hz. Thanks to its lightweight (15 g) and robust housing, the ReSense module is particularly well suited for clinical applications. In addition, the on-board clock of multiple modules can be synchronized temporally via a custom-built USB base station.

#### Indirect calorimetry

EE was assessed using a portable metabolic cart (Oxycon mobile, Carefusion, Hoechberg, Germany; Figures 1A,D,E). This system consists of a facemask, a turbine to assess flow as well as O_2_ and CO_2_ analysers. The data exchange unit as well as the sensor box were fixed on the participant's back via a harness. Data were recorded continuously breath-by-breath (i.e., one data point per breath) and synchronized offline with ReSense measurements. EE was derived from the O_2_ consumption and CO_2_ production using the proprietary software JLAB (Carefusion, Hoechberg, Germany). The system was calibrated according to the manufacturer's recommendation, 30 min prior to and immediately after each session in order to ensure high accuracy. Additionally, HR and blood oxygen saturation were measured by infrared technology, using an ear clip that was connected to the same system.

#### Bioelectrical impedance analysis (BIA)

BIA was used to determine fat mass (FM) and fat-free mass (FFM) of each subject. Based on FM and FFM, an additional reference value for the resting energy expenditure (REE) was calculated. For BIA measurements, a signal electrode as well as a measurement electrode were attached at each hand and foot and connected to the BIA device (AKERN BIA 101 system, SMT medical, Würzburg, Germany). FM and FMM were calculated using the proprietary software (BodyComposition, MEDI CAL HealthCare GmbH, Karlsruhe, Germany).

### Clinical assessments

Prior to the experiment, three standard clinical assessments were conducted to gather information on the NLI, the severity of the lesion, and the independence of the SCI subjects. The ISNCSCI protocol was used to assess the NLI as well as the completeness of the lesion (25). The Spinal Cord Independence Measure III (SCIM III) is a questionnaire containing 19 items, which was used to assess the level of independence in daily life (26). The total score, ranging from 0–100, as well as the scores of the three subdomains self-care (range 0–20), respiration and sphincter management (0–40), as well as mobility (0–40) were included separately in the analysis. The Graded Redefined Assessment of Strength, Sensibility and Prehension (GRASSP) was used to capture motor and sensory function and functional task performance of the upper extremities. As the GRASSP assesses sensation, strength, qualitative and quantitative grasping through a series of five examinations, the total score and the individual sub-scores of the five examinations were later included separately in the analysis (27, 28).

### Tasks

Each subject had to perform 12 different physical activities out of a set of 24 possible activities. These activities were divided into three activity classes based on measured EE, subjectively perceived exertion, and the amount of distance traveled. The “low-intensity” activity class included the following activities: rest (lying on a bed), watching TV, reading, doing crossword puzzles, playing cards, riding an elevator, playing with a tablet PC, writing, computer work, and passive wheeling (i.e., when the wheelchair was pushed by someone else). The “high intensity” class included the following activities: washing dishes, hanging out the laundry, using a handbike ergometer (30 W), playing table tennis, and weight lifting. The last class was called “wheeling” and included activities involving wheelchair self-propulsion. These activities included completing a wheelchair skill parcour (including a slalom with nine cones, four curbs of 3–8 cm height, and a ramp with an inclination of 8%), wheeling at different speeds (2, 3.5, 5, 6.5 km/h and self-chosen), wheeling uphill (inclination 2.6%), wheeling downhill (inclination 2.6%), and wheeling on a wheelchair ergometer.

### Protocol

Participants came to the Balgrist University Hospital for a single session of ~5 h in the morning after an overnight fast of at least 10 h. First, participants were informed about the experimental procedure, and the 12 pseudo-randomly selected tasks were explained in detail. Subsequently, body composition was assessed by use of BIA, height was measured while the subject was lying on the bed, and weight was measured with a wheelchair scale. Participants were equipped with one sensor module at each wrist, one module was fixed at the chest (approximately at the sternum) and an additional module was fixed to one wheel of the wheelchair. Participants used their own, individually adapted wheelchair for the entire duration of the study. Thereafter, the sensor modules were time-synchronized with the camera, the indirect calorimeter, and the HR monitor.

The first part of the experiment consisted of 20 min of rest, lying on a bed for assessment of REE, followed by a standardized breakfast equivalent to 30% of a participant's calculated daily EE. The second part of the experiment started at least 90 min after the end of breakfast, when EE had returned to baseline values. First, participants lay on a bed for 20 min. This was considered the REE measurement under the non-fasted condition (first task). Subsequently, participants performed eleven 8-min tasks, selected pseudo-randomly from the set of 24 tasks, with a minimum of 5 min between two consecutive tasks. The pseudo-random selection ensured that at least two tasks from each activity class were selected and that each task was performed approximately equally often across all subjects. The 8-min activities were ordered according to the expected intensity of the tasks, starting with the least intense task. After each task, subjects were asked to rate their perceived exertion on an 11-point numeric rating scale (0 = “no exertion,” 10 = “maximum exertion”).

Video recordings were taken during the entire experiment (GoPro Hero HD 2, Go Pro Inc., San Mateo, CA, USA) in order to verify all activities retrospectively. In case subjects were unable to start the experiment in the morning, a shortened version of the protocol was provided, i.e., subjects came to the clinic at least 2 h after the last food intake. After explanation of the study and signing the consent form, the experiment started with the assessment of body composition, height, and weight, followed by 20 min of REE measurement in the non-fasted condition. Afterwards, subjects followed the same protocol as described above.

### Data analysis

The complete data processing, statistical analysis, as well as the training of the k-nearest neighbors (kNN) classifier and ANNs were performed using MATLAB 2014a (The MathWorks, Natick, MA, USA). All processing steps were conducted offline.

**Figure 2:**
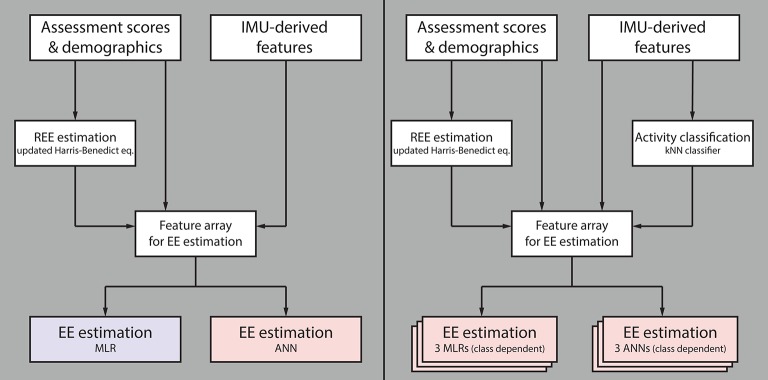
Flow chart summarizing the different evaluated algorithms. The first algorithm for estimating energy expenditure (EE) consists of a multiple linear regression (MLR) model and the second algorithm of an artificial neural network (ANN); both use features derived from IMU data, participant demographics and clinical assessment scores, as well as previously estimated resting EE (REE) as model input **(Left)**. The third algorithm consists of three independent MLRs (one per activity class), and the fourth of three independent ANNs, both preceded by a k-nearest neighbors (kNN) classifier **(Right)**. The two latter algorithms use the same model inputs as algorithms one and two, without preceding kNN classifier. Note that the blue model requires only limited computational power compared to the red ones.

In total, four different algorithms were designed, evaluated (Figure 2) and compared against algorithms described in the literature. The first algorithm is of low complexity, which requires only limited computational power and could therefore be implemented directly on an activity tracker for on-line analysis and subject feedback. This algorithm consists of a multi-linear regression (MLR) model which uses different statistical features derived from the IMU data and previously estimated REE as predictors. The second algorithm is a more complex approach requiring more computational power. The algorithm consists of an ANN using features derived from the sensor data and the estimated REE as predictors. The third (MLR based with prior classification) and the fourth (ANN based with prior classification) algorithms are motivated by the work of Staudenmayer et al. (16). This group showed that prior classification into different activity classes before the EE estimation increases the estimation accuracy significantly. Therefore, the third and fourth algorithms consist of three parts. In the first part, the REE is estimated from demographic data; in the second part the different activities are classified into the three activity classes; in the third part, the EE is estimated using different MLRs or ANNs, respectively, for each of the classes. All processing steps are explained in the following paragraph.

#### Pre-processing

In order to ensure that all IMU data consisted of the same number of samples and that they were temporally aligned, the recordings from the ReSense modules were resampled at 50 Hz using a cubic spline interpolation function. Afterwards, IMU recordings were synchronized with the OxyconMobile data and the video recordings, using time stamps, which were aligned at the beginning of the experiment. The acceleration signals were filtered using a 2nd order Butterworth high-pass filer with a cut-off frequency of 0.25 Hz in order to remove the static acceleration component due to gravity. Gyroscope data were filtered with the same high-pass filter. The altitude data was filtered using a 2nd order Butterworth low-pass filter with a cut-off frequency 0.2 Hz.

#### Labeling and segmentation

Data were labeled using temporal markers from the OxyconMobile, the IMU modules, and from the video recordings. For the REE measurements, the mean of a 4-min window (min 14–18) was taken. For each of the individual activities, the last 4 min of each activity were segmented in windows of 1 min without overlap. Taking the last 4 min ensured that the EE had reached a steady state. After visual inspection of the data, 1,324 windows remained, which were later included in the development of the different models. As HR data was partly missing for some subjects, all analysis involving the HR was only based on 897 windows (67.8%).

#### Feature calculation

Features were calculated from the processed acceleration signal containing the dynamic component, from the gyroscope data and the altitude signal for each window. All statistical features derived from the accelerometer and gyroscope data were calculated from the respective magnitudes in order to ensure that the orientation of the sensors, and therefore in which orientation the sensor is placed, had no influence on the final algorithm. Statistical features derived from the sensor data were based on previously used features in activity classification studies (29–39). Only features from the time domain were taken for further analysis as we have shown that in “real world” applications, frequency domain features usually do not provide useful information (40). In addition, some high level features were included: AC (41), total distance traveled and distance traveled actively (40), altitude difference within one epoch, altitude variance within one epoch, and time above acceleration magnitude threshold (empirically chosen). In order to test whether the inclusion of HR improves the overall accuracy of EE estimation, as shown by Nightingale et al. (42), mean HR, resting HR and the difference between mean HR and resting HR were included as additional features. Finally, features extracted from demographics as well as from clinical assessments were included, namely age, height, weight, gender, AIS score (A = 4, D = 1), injury level (C1 = 1, L5 = 25), GRASSP sub-scores and SCIM III sub-scores.

#### REE estimation

Five different MLR models were developed for the estimation of the REE. The different MLR models have all the following basic form:

$$\begin{array}{l} REE=\alpha +\overset{n}{\underset{i=1}{\sum }}\beta_{i}\cdot F_{i} \end{array}$$

with α representing the intercept, and β_*i*_ representing the regression coefficient of the feature *F*_*i*_. All MLR models included the independent variables height, weight, age and gender. In addition, different clinical scores were also included as independent variables, namely total SCIM III score, completeness, NLI, AIS score, and motor score of the ISNCSCI assessment. The MLR was computed by minimizing the sum of squared relative errors according to the method described by Tofallis (43). Moreover, ANNs based on the same features were trained in order to reveal more complex and non-linear relationships. Readers not familiar with ANNs can refer to the work of Basheer and Hajmeer (44). As one hidden layer is usually sufficient in most applications, all ANNs had a single hidden layer with five sigmoid neurons (44). The initial weights were chosen by the Nguyen-Widrow layer initialization function and the Levenberg-Marquardt backpropagation algorithm was used to train the ANNs (45, 46). As the actual output can vary depending on the initial weights, 100 ANNs were trained per iteration and the mean outcome was used for further analysis. The performance was analyzed using the leave-one-subject-out cross-validation, resulting in 30 iterations. The MAE in percent was chosen as criterion for the MLRs and ANNs. A detailed overview of the models and features included in this study can be found in Table 2. In addition to the REE estimation models described above, three well established estimation models, which have been developed for the able-bodied population, were evaluated with the data from this study: the Harris-Benedict equation, the updated Harris-Benedict equation and the Mifflin-St. Jeor equation (47–49). Finally, the REE was also estimated from the BIA measurement using the BIA software BodyComposition Professional (Medi Cal HealthCare GmbH, Karlsruhe, Germany).

**Table 2:** Results of the REE estimation based on BIA measurement, models known from the literature (°), and MLR and ANN models developed in this study.

	**Mean absolute error**				**Mean signed error**		**Max error**	
**Model description**	**[%]**	**[kcal/day]**	**tetraplegic [%]**	**paraplegic [%]**	**[%]**	**[kcal/day]**	**[%]**	**kcal**
BIA^*^	23.3 ± 12.2	409.7 ± 255.7	23.5 ± 12.2	23.2 ± 12.5	−22.5 ± 13.7	−400.7 ± 270.2	48.3	956.1
Harris-Benedic°	14.2 ± 7.9	229.8 ± 129.1	13.9 ± 7.9	14.5 ± 8.2	1.9 ± 16.4	0.5 ± 267.1	26.7	528.2
updated Harris-Benedict°	14.2 ± 7.8	227.8 ± 123.1	13.5 ± 7.4	14.6 ± 8.2	2.3 ± 16.3	7.0 ± 262.4	25.9	511.4
Mifflin-St. Jeor°	15.1 ± 11.8	256.1 ± 204.9	16.7 ± 12.8	14.1 ± 11.4	−8.1 ± 17.5	−169.5 ± 283.1	34.6	648.8
**MULTIPLE LINEAR REGRESSION**								
h, w, a, g	15.7 ± 12.0	250.3 ± 182.6	15.0 ± 8.6	16.1 ± 13.8	1.3 ± 19.9	63.3 ± 306.7	48.7	810.5
h, w, a, g, SCIM III	16.6 ± 15.5	262.4 ± 227.6	13.2 ± 8.0	18.7 ± 18.5	−0.4 ± 22.9	37.7 ± 348.8	67.8	933.3
h, w, a, g, AIS	16.3 ± 11.3	261.5 ± 175.1	16.2 ± 7.1	16.3 ± 13.5	1.2 ± 20.0	63.2 ± 312.0	48.5	807.3
h, w, a, g, NLI	16.4 ± 11.5	264.2 ± 175.7	15.1 ± 8.4	17.2 ± 13.2	0.9 ± 20.3	57.9 ± 315.7	48.9	814.4
h, w, a, g, motor score	16.6 ± 12.7	264.1 ± 190.8	16.0 ± 9.3	16.9 ± 14.6	1.2 ± 21.0	64.9 ± 322.9	49.2	818.2
**ARTIFICIAL NEURAL NETWORK**								
h, w, a, g	16.2 ± 12.2	248.9 ± 156.5	14.3 ± 8.3	17.4 ± 14.1	2.7 ± 20.4	−6.6 ± 297.7	32.2	535.7
h, w, a, g, SCIM III	14.2 ± 12.2	215.7 ± 160.0	10.0 ± 5.8	16.7 ± 14.4	2.8 ± 18.7	4.2 ± 271.5	39.7	660.4
h, w, a, g, AIS	17.0 ± 11.0	263.0 ± 137.6	15.4 ± 7.4	18.0 ± 12.9	2.4 ± 20.4	−12.6 ± 300.6	49.7	528.5
h, w, a, g, NLI	16.6 ± 11.0	256.5 ± 136.5	14.6 ± 6.9	17.9 ± 12.9	2.7 ± 20.0	−5.8 ± 294.5	33.3	554.3
h, w, a, g, motor score	15.8 ± 12.3	240.1 ± 155.0	12.2 ± 8.4	17.9 ± 14.0	2.9 ± 20.0	−1.2 ± 289.4	54.4	579.0
h, w, a, g, AIS, NLI	17.4 ± 11.7	266.8 ± 140.5	14.4 ± 8.2	19.2 ± 13.2	2.9 ± 21.0	−5.7 ± 305.6	52.3	557.0

h, height; w, weight; a, age; g, gender;

* *For the REE estimation based on the BIA measurement only N = 28 subjects were included*.

#### Activity classification

In order to classify the different windows into one of the previously described activity categories “low-intensity,” “high-intensity,” and “wheeling,” a kNN classifier with *k* = 10 and a squared inverse distance weight was used. A total of *n* = 1384 windows were used to train the kNN classifier. Three features were selected for the kNN classifier, namely the AC of the wheel sensor as well as the root mean square (RMS, right wrist) and the median (left wrist) of the angular velocity magnitude. Among all feature combinations, the combination with these three features showed the best classification accuracy. In order to evaluate the performance of the kNN classifier, the leave-one-subject-out cross-validation method was used for the analysis. This resulted in a total of 30 iterations, one per subject. The percentage of correct classified windows was taken as a criterion to optimize the classifier.

#### EE estimation

In total four different estimation models were designed for the activity dependent EE not including the HR. An MLR model and an ANN model were designed where the activity-dependent EE was estimated using IMU data and the estimated REE as predictors. In order to see if a prior classification into different activity classes increases the estimation accuracy, additional MLR and ANN based models were designed. Thereby each activity class had a separate MLR or ANN estimation model. In order to see how the classification accuracy of the previously mentioned kNN classifier influences the final EE estimation, the MLR and ANN models with prior activity classification were evaluated (i) assuming 100% correct classification and (ii) with the classes estimated by the kNN classifier. Similar to the REE estimation using MLR, the MLRs for the activity-depended EE estimation were computed using the sum of squared relative errors (43). The MLR model without prior classification used seven predictors in total (right wrist IMU: mean acceleration magnitude, kurtosis of the angular velocity magnitude, altitude difference; left wrist IMU: RMS of the acceleration magnitude; chest IMU: RMS of the angular velocity magnitude; wheel IMU: variance of the angular velocity magnitude; REE). The MLR model with previous classification used for the MLR of the “low-intensity” class the same features as predictors as mentioned before, the “wheeling” class used one predictor less, specifically, the RMS of the acceleration magnitude of the left wrist IMU. The “high intensity” class used slightly different features as predictors (right wrist IMU: altitude difference; left wrist IMU: RMS of the acceleration magnitude; chest IMU: median of the acceleration magnitude, RMS of the angular velocity magnitude; wheel IMU: kurtosis of the acceleration magnitude, variance of the angular velocity magnitude; REE). The ANNs trained for the estimation of the activity-dependent EE had the same design as the ANNs trained for the REE estimation. This means that all ANNs had one hidden layer with five sigmoid neurons. The Nguyen-Widrow layer initialization function was used to choose the initial weights and the Levenberg-Marquardt backpropagation algorithm was used to train the ANNs. Here again, 100 ANNs were trained per excluded subject and the mean outcome was used for further analysis. The ANN without prior activity classification used six features as input, namely the mean acceleration magnitude (right wrist IMU), AC (left wrist IMU), AC (chest IMU), altitude difference (chest IMU), distance traveled, and REE. The ANN model with prior activity classification had different features as inputs for every ANN. The ANN for the activity class “low intensity” had seven inputs, namely the mean acceleration magnitude (right wrist IMU), kurtosis of the angular velocity magnitude (right wrist IMU), altitude difference (right wrist IMU), RMS of the angular velocity magnitude (chest IMU), weight, gender, and estimated REE. The ANN for the “high intensity” class used five features as input, namely AC (right wrist IMU), mean acceleration magnitude (left wrist IMU), mean angular velocity magnitude (chest IMU), AC (left wrist), and estimated REE. The ANN for the “wheeling” class had six inputs: RMS of the acceleration magnitude (right wrist IMU and left wrist IMU), AC (chest IMU), mean angular velocity magnitude (wheel IMU), AC (left wrist), and estimated REE. In order to test if the inclusion of HR improves the estimation, all models (MLR and ANN based) with prior activity classification were trained again but this time including the difference between measured HR and resting HR as additional predictor. Note that only 897 windows were available for the training of the models including HR. All developed models (MLR, ANN, with and without HR) were evaluated using the leave-one-subject-out cross-validation method. This resulted in a total of 30 iterations for each developed model.

#### Establishing reference values for a healthy lifestyle

For a healthy lifestyle, different reference values exist for the able-bodied population. The most well-known reference value for a healthy lifestyle is probably the 10,000 steps a day reference (equivalent 300 kcal/day) (50, 51). However, this reference value is controversial and other research groups and institutions have proposed lower daily step-goals (52). Furthermore, other activity goals, which are not directly related to the number of steps, have been proposed.

The U.S. Department of Health and Human Services for example, suggests 150 min of moderate physical activity per week (e.g., 5 × 30 min) or 75 min of vigorous physical activity per week (53). This is similar to what the American College of Sport Medicine (ACSM) suggested in their updated recommendations from 2007, where 30 min of moderate physical activity on 5 days a week or 20 min of vigorous physical activity on 3 days per week is suggested to promote a healthy lifestyle (54). Blair and colleagues stated in their work, that 30 min of moderate intensity activity per day provides substantial benefits, but 60 min of moderate intensity activities per day would be ideal (55). These 60 min of moderate (to vigorous) activity per day are also what is recommended by Wilson and colleagues in order to prevent weight gain (56). Sixty minutes of moderate to vigorous activity per day results in an increase in EE by 150–200 kcal/day. In order to establish values for a healthy lifestyle in the wheelchair bound SCI population, we therefore investigated which daily distance traveled in wheelchair would result in 150, 200, and 300 kcal/day. In order to translate this recommendation for a healthy lifestyle into a daily distance to travel by wheelchair, the daily average speed has to be taken into consideration. Since the average wheeling speed in the SCI population was reported to be 1.7–2.3 km/h (57, 58), the EE measured in the wheeling task at 2 km/h was used. On the other hand, there are recommendations based on time spent in activities of moderate (to vigorous) intensity. Therefore, we estimated the distance to travel and the corresponding EE based on the recommendation corresponding to 30 and 60 min of wheeling at moderate intensity.

#### Performance analysis and statistics

The performance of the REE and EE estimation models were analyzed in terms of MAE in percent and mean signed error (MSE) in percent. The performance of the kNN classifier was analyzed using overall classification accuracy in percent and in addition the sensitivity of the different classes was computed (40). In order to compare different activities, the metabolic equivalent of task (MET) was calculated using an adapted formula for the SCI population (59), where 1 SCI MET is equivalent to 2.7 mL O_2_·kg^−1^·min^−1^ in contrast to the formula for the able-bodied population, where 1 MET is equivalent to 3.5 mL O_2_·kg^−1^·min^−1^ (60). In order to demonstrate the relationship between measured SCI MET and perceived exertion (numeric rating scale), a Spearman rank correlation was used. The significance level for all statistical analyses was set to *p* = 0.05.

## Results

### REE estimation

An overview of all REE estimation models is presented in Table 2. The estimates are compared in terms of MAE, MSE and maximal error. Both Harris-Benedict equations as well as the ANN with height, weight, age, gender and total SCIM III score performed best in terms of MAE (14.2%). Generally, all models overestimated the REE, which is reflected in the positive MSE. Only the estimation with the BIA, the Mifflin-St. Jeor equation and the MLR model including height, weight, age, gender, and total SCIM III score showed an underestimation of the true EE value. The MAE in percent for men was always lower than the MAE for women, except for the BIA estimation where an MAE for men of 24.0 ± 11.5% and MAE for women of 17.8 ± 19.2% were found. By way of comparison, the MAE resulting from the updated Harris-Benedict equation was 13.0 ± 7.2% for men and 25.0 ± 7.2% for women.

### Activity classification

**Figure 3:**
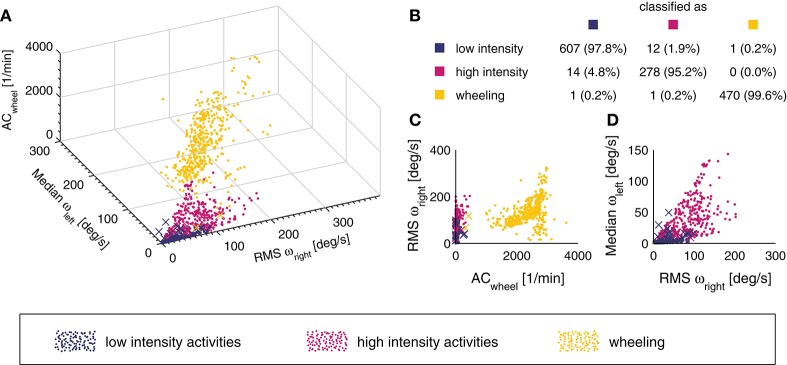
3D scatter plot of the different activity classes for the three features used **(A)**. A point represents a correct prediction and a cross a false prediction. In addition, the confusion matrix for the entire data set (*n* = 1,384 windows) is represented in subplot **(B)**. The overall classification accuracy was 97.9%. In order to illustrate how the different activity classes can be separated, two additional projections of the 3D scatter plot are presented **(C,D)**.

The overall classification accuracy of the kNN classifier was 97.9%. An overview of the classification accuracy of each individual activity class can be found in Figure 3B. In addition, a 3D scatter plot (Figure 3A) and two 2D scatter plots (Figures 3C,D) are presented in order to visualize the separation of the different activity classes. The sensitivity for the individual activities was generally high, with a range of 81.8–100% and a median of 100%.

### EE estimation

**Figure 4:**
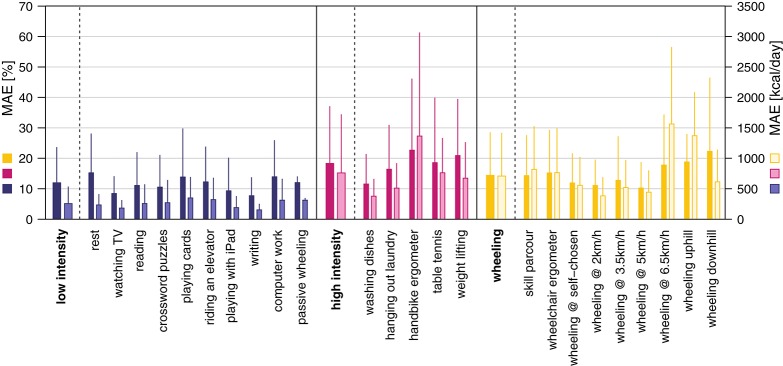
Mean absolute error (MAE) for the EE estimation using the ANN model with prior activity classification. The overall MAE was 14.4 ± 5.3%. The MAE in percent for the single activities and classes is presented in dark colors and the MAE in kcal is presented in bright colors.

ANN and MLR models were based on a total of 1,324 windows. An overview of the EE estimation accuracy of all models can be found in Table 3. Overall, the ANN model where the activity was previously classified into classes by the kNN classifier showed the lowest overall MAE for the EE estimation with 14.4 ± 5.3% (Figure 4). The MAE of the different activity classes for the previously mentioned EE estimation model was 11.8 ± 6.1% for the “low intensity” class, 19.2 ± 11.7% for the “high intensity” class, and 14.4 ± 6.8% for the “wheeling” class. Assuming a classification accuracy of 100% (ANN class known) for the kNN classifier, this would only result in a marginally better overall MAE (14.1 ± 5.4%) for the EE estimation. The MAE for the different activity classes was 11.8 ± 6.0% for the “low intensity” class, 17.6 ± 11.7% for the “high intensity” class, and 14.2 ± 6.9% for the “wheeling” class. The MLR model (with prior kNN classification) profited from the inclusion of the HR. The overall MAE for the EE estimation improved from 16.0 ± 6.2 to 14.9 ± 4.8% when including the HR. The MAE for the different classes was 13.7 ± 6.8% without HR and 13.8 ± 6.1% with HR for the “low intensity” class, 19.0 ± 10.7% without HR and 17.4 ± 9.4% with HR for the “high intensity” class, and 17.9 ± 9.3% without HR and 17.2 ± 10.9% with HR for the “wheeling” class. In contrast to the MLR model, the ANN model (with prior kNN classification) did not benefit from the inclusion of the HR. The overall MAE for the EE estimation with the ANN model was 12.9 ± 4.7% without HR and 13.6 ± 5.4% with inclusion of the HR. Looking at different classes showed that the MAE was 10.8 ± 5.9% without HR and 12.3 ± 6.9% with HR for the “low intensity” class, 18.6 ± 13.2% without HR and 17.3 ± 9.8% with HR for the “high intensity” class, and 14.2 ± 8.6% without HR and 15.1 ± 12.2% with HR for the “wheeling” class.

**Table 3:** Evaluation of the different models developed as a part of this study.

	**Mean absolute error**				**Mean signed error**		**Max error**	
**Model description**	**[%]**	**[kcal/day]**	**tetraplegic [%]**	**paraplegic [%]**	**[%]**	**[kcal/day]**	**[%]**	**kcal**
***N*** = **1,324**								
MLR general	15.3 ± 4.8	592.7 ± 270.9	16.4 ± 4.7	14.6 ± 4.9	−2.9 ± 11.1	−266.2 ± 444.4	27.3	1180.1
MLR class known	15.0 ± 4.7	579.2 ± 255.1	15.9 ± 4.2	14.5 ± 5.1	−2.5 ± 10.5	−233.0 ± 416.7	25.7	1103.8
MLR class estimated	15.2 ± 4.7	584.0 ± 253.6	16.3 ± 4.0	14.5 ± 5.0	−2.5 ± 10.5	−235.0 ± 415.4	25.8	1108.9
ANN general	17.3 ± 6.8	606.5 ± 225.1	18.2 ± 6.6	16.8 ± 7.1	5.4 ± 13.4	20.3 ± 489.9	26.8	1118.0
ANN class known	14.1 ± 5.4	513.6 ± 201.3	14.9 ± 4.7	13.6 ± 5.8	3.3 ± 9.8	2.9 ± 379.8	23.9	980.0
ANN class estimated	14.4 ± 5.3	524.7 ± 205.2	15.5 ± 4.7	13.8 ± 5.7	3.5 ± 9.9	−1.3 ± 384.4	24.4	1011.6
**N** = **897 (HR analysis)**								
MLR class estimated	16.0 ± 6.2	576.9 ± 281.6	16.9 ± 4.7	15.6 ± 7.0	2.9 ± 12.6	−221.8 ± 437.9	35.3	1406.9
MLR class estimated with HR	14.9 ± 4.8	513.9 ± 193.5	16.4 ± 4.5	14.1 ± 4.8	−1.8 ± 12.3	−154.2 ± 426.2	24.6	961.0
ANN class estimated	12.9 ± 4.7	419 ± 141.4	12.8 ± 3.1	12.9 ± 5.4	3.4 ± 8.2	35.1 ± 284.6	19.4	659.7
ANN class estimated with HR	13.6 ± 5.4	445.8 ± 160.7	14.5 ± 5.5	13.0 ± 5.5	3.0 ± 10.2	14.6 ± 336.4	23.0	816.0

*The analysis including the HR was based only on N = 897 windows*.

**Figure 5:**
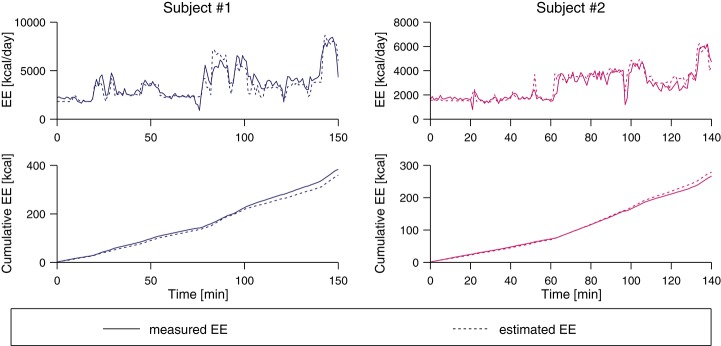
Pre-validation of two subjects using the class-dependent ANN algorithm. The two top plots show the measured EE from the indirect calorimetry and the estimate based on the IMU data, and the bottom plots show the cumulative value of the estimated and the measured EE value. At the end of the measurement period, the real EE was underestimated by 6.1% for subject #1 and overestimated by 4.6% for subject #2.

The evaluation of the two 2.5 h-measurements to validate the algorithm under real-world conditions is shown in Figure 5. For subject #1, the real EE was underestimated by 23.2 kcal (6.1%) while for subject #2, the real value was overestimated by 12.2 kcal (4.6%).

### Energy cost of physical activities

**Figure 6:**
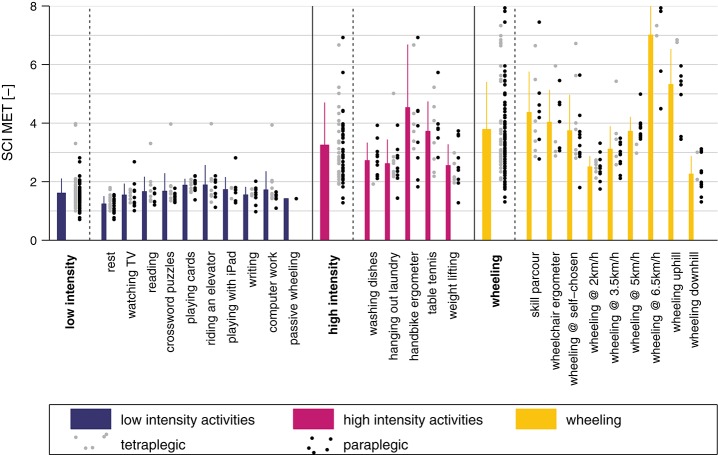
SCI MET presented for all activities and classes. The gray dots to the right of each bar represent the values for the tetraplegic subjects and the black dots the values for the paraplegic subjects. The bars represent the mean of the pooled data including paraplegic and tetraplegic subjects.

**Figure 7:**
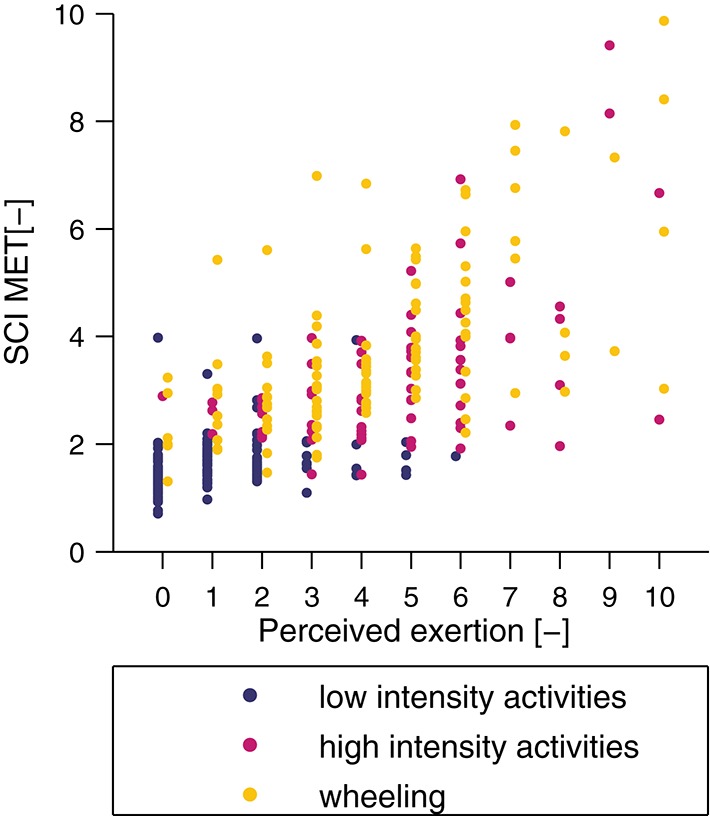
Correlation between perceived exertion, assessed with a numeric rating scale, and measured SCI MET. The correlation coefficient was *R* = 0.73 (*p* < 0.001).

The metabolic cost of each single activity and the three activity classes is presented in Figure 6. The mean SCI MET for the “low-intensity” class was 1.6 ± 0.5 and all activities were below 2 SCI MET, which is considered as light intensity activity (61). The mean SCI MET for the “high-intensity” class was 3.2 ± 1.5 and three activities of this class are considered as light intensity activities and two as moderate intensity activities. For the “wheeling” class the mean SCI MET was 3.8 ± 1.6 and, according to Pate and co-workers, two activities would be classified as light intensity activities, six as moderate intensity activities, and one as vigorous intensity activity (61). In order to show the relationship between measured SCI MET and perceived exertion, a correlation (*R* = 0.73, *p* < 0.001) is presented in Figure 7.

### Recommendations of healthy lifestyle

The conversion of the recommendations for a healthy lifestyle in the able-bodied population to daily goals in the wheelchair-bound SCI population can be found in Table 4.

**Table 4:** Recommendations for the able-bodied population converted to EE during moderate activity, and to distance to travel for the wheelchair-bound SCI population.

		**EE**	**Speed**	**REE measured**	**EE measured**	**Extra EE measured**	**Distance**	**Time needed**
**Recommendation**	**Reference**	**[kcal/task]**	**[km/h]**	**[kcal/day]**	**[kcal/day]**	**[kcal/day]**	**[km]**	**[min]**
10,000 steps of able-bodied	50, 51	300	2.0	1,650	3,430	1,780	**8.1**	**243**
150 kcal of additional EE	56	150	2.0	1,650	3,430	1,780	**4.0**	**121**
200 kcal of additional EE	56	200	2.0	1,650	3,430	1,780	**5.4**	**162**
30 min of moderate activity	55	**54**	3.5	1,644	4,255	2,611	**1.8**	30
	55	**61**	5.0	1,559	4,502	2,943	**2.5**	30
60 min of moderate activity	56	**109**	3.5	1,644	4,255	2,611	**3.5**	60
	56	**123**	5.0	1,559	4,502	2,943	**5.0**	60
Recommendations based on this study (following ACSM recommendation)		**74**	**5.0**	**1,559**	**4,502**	**2,943**	**3.0**	**36**
Additional recommendations based on this study		**147**	**5.0**	1,559	4,502	2,943	**6.0**	**72**
	^*^	**92**	**3.5**	1,644	4,255	2,611	**3.0**	**51**
	^*^	**111**	**2.0**	1,650	3,430	1,780	**3.0**	**90**

* *The activities used for this recommendation cannot always be classified as an activity of moderate intensity. Note that values in bold are calculated or estimated from directly measured or predefined values given in the table*.

## Discussion

The main aim of this study was to develop and validate an algorithm for estimating EE from IMU data applicable in a real-world situation in individuals with SCI. We present a highly accurate method to estimate ADL-dependent EE from IMU recordings. Most importantly, we provide reference values for wheelchair-bound SCI subjects to promote and coach a healthy lifestyle, which could be beneficial for reducing the risk of cardiovascular diseases.

REE reflects the energy used to maintain vital functions at room temperature. To account for its large contribution [~65% in the able-bodied population (62)] to total daily EE, it was deemed important to accurately estimate the REE. Also, at a later stage, the estimated REE was used as predictor for the activity-dependent EE estimation models. As expected, the BIA-based model showed the worst REE estimation in terms of MAE, MSE, and maximum error, and is generally known to be very population specific (63). Therefore, a separate BIA estimation model for SCI would be required. The three well-established REE estimation equations, namely the Harris-Benedict equation, the updated Harris-Benedict equation, and the Mifflin-St. Jeor equation performed equally well. The MAE for REE estimation using the updated Harris-Benedict equation has been reported to be around 14% for the able-bodied population (49). This compares favorably to 14.2 ± 7.9% for the SCI data in the present study.

Here, we also investigated whether and how REE estimation models could be improved by considering clinical scores. However, the inclusion of the AIS score did not improve the models and is likely too unspecific in describing the extent of impairment. Also, including the level of injury or the motor scores of the ISNCSCI did not improve the estimation accuracy significantly. The only clinical score improving the MAE of the REE estimation was the SCIM III total score, although it only improved the ANN-based model. In general, all REE estimation models that were developed in this study were based on the data of only 30 subjects. This number is clearly too small to build a general model for this heterogeneous population of SCI subjects. For this reason, subsequent analyses were performed using the updated Harris-Benedict equation for REE estimation.

In the able-bodied population, the type of activity performed by a subject was shown to be of great importance when establishing models to estimate EE (16). In order to obtain a generalizable EE estimation model, we chose to split the different activities into three broader activity classes. The overall classification accuracy of the kNN classifier was slightly better than previous comparable models developed for the SCI population, which can most likely be explained by the fact that we included only three activity classes, whereas Hiremath and co-workers included four and seven activity classes, respectively (22, 64). The kNN classification has proven to be an appropriate approach for activity classification in the able-bodied population as well as in neurological conditions other than SCI (31, 36, 38, 39). The performance of our kNN classifier was excellent, with an overall classification accuracy of 97.9%. The activity class “wheeling” had only 2 out of 472 misclassified events. Already a single feature, namely AC of the wheel sensor, was enough to classify the aforementioned class (Figure 3C). The “low intensity” and “high intensity” class had 13 and 14 misclassified events. In the “low intensity” class, the activity “playing cards” showed the overall worst sensitivity with 85.3%. This result can be explained by the fact that even if playing cards is considered as low intense activity, it can include extended periods of faster and more intense arm movements. However, in only 2 out of 24 activities, the presented kNN classifier had a sensitivity lower than 90%. Thus, the presented kNN classifier is accurate enough to be used for the activity classification of the final EE estimation algorithm. Furthermore, since the kNN classifier classifies activities into activity classes (i.e., groups of activities) and not into single activities, this approach may be generalizable to other activities and applications.

The MAE of the activity-dependent EE estimation ranged from 14.1% up to 17.3%, when the HR information was not included. However, we have to take into account that the model reaching a MAE of 14.1% assumed a perfect classification into the different activity classes. In general, the overall MAE is in the range of other accelerometer or IMU based models developed for the SCI population although those studies included fewer activities (21, 22, 42). Recently, Hiremath and coworkers presented an EE estimation model which was developed using recordings from 20 different activities, achieving a MAE of 25% (23). A possible explanation for the different results might be that Hiremath and coworkers used linear regression models, which do not account for non-linear relationships between accelerometer measurements and EE (23). MAE in the present study was always highest for the “high intensity” class, which can be explained by the fact that weight-loading activities, such as the handbike ergometer and weight lifting, have been included in this class. In fact, a similarly decreased estimation accuracy for activities with external load has already been reported previously in the able-bodied population (13, 18). In order to obtain the best EE estimate for each class, different features where used for the different classes. Interestingly, all models developed in this study, whether MLR or ANN based, included features derived from the accelerometer data as well as from the gyroscope data. This is in contrast to what Moncada-Torres and co-workers have shown, where features derived from the gyroscope data did not provide useful information for activity classification (39). This discrepancy can be explained by the fact that, in our study, the algorithms were used to estimate a continuous value of EE, while in the study by Moncada-Torres the algorithms were used to classify activities (39). The inclusion of altimeter-based features in the final algorithm was not surprising, as it has already been shown that accelerometer and altimeter are a good combination to estimate EE in activities with altitude changes (65). Two features based on demographic data were further included, namely weight and gender. These two features were only selected in the class-dependent model and only for the “low intensity” class. Again, the exclusion of features based on demographic data may be explained by the fact that they are already represented in other features, especially in the REE. The comparison between MLR and ANN-based models showed that the MLR-based model performed better when no previous activity classification occurred, and the ANN-based model performed better for the class-dependent model.

The inclusion of the HR showed a slight improvement for the MLR-based model. Thereby, the overall estimation improvement comes mainly from the improvement in the “high intensity” class. This might be due to the fact that the addition of the HR can, to a certain extent, improve the EE estimate of weight loading activities. The validity of combining accelerometer and HR measurements in the SCI population to estimate EE by using linear models has already been shown by Nightingale et al. (42). Our non-linear approach based on the ANNs did not benefit from the inclusion of the HR, and the MAE increased when adding the HR as additional predictor. While the MAE decreased for the “high intensity” class, it increased slightly for the “wheeling” motion class and was negligible for the “low intensity” class. In fact, the change in HR might not necessarily result from a change in the intensity of an activity, especially in the “low intensity” class, as HR is known to be influenced also by emotion, stress or other factors which are most visible at rest and during low intensity exercise (66). During the assessments in the present study, activity-independent factors potentially influencing HR were minimized. Hence, the application of the ANN model with HR as additional feature would most likely result in even higher estimation errors under “real-world” conditions. Therefore, we suggest using the class-dependent ANN model without HR for future applications.

Based on the insights from this study and existing literature for the able-bodied population, we seeked to propose activity-related recommendations for a healthy lifestyle in the SCI population. For subjects with SCI, activity recommendations were translated into daily distance traveled in a manual wheelchair. Since translations from able-bodied to SCI are based on the EE recordings of the present study, we assured that the different SCI MET values in the literature matched the SCI MET values of this study. Collins and co-workers investigated the metabolic cost of 27 different physical activities in 170 adults with SCI (59). Six activities, namely desk work, laundry, washing dishes, weight lifting, table tennis, and hand bike ergometer were also included in our study. The measured mean SCI MET of these activities was indeed in the same range as was reported previously, except for the hand bike ergometer activity, where mean SCI MET was 4.5 in the present study compared to values between 3.37 and 3.83 reported by Collins et al. (59). Possibly, this difference can be explained by differing angular velocities of the hand bike ergometer between the two studies. Further agreement between the SCI MET of this study and the literature can be found in the study of Hiremath et al. (23). For the four common activities of both studies, namely arm ergometry, deskwork, resting and propulsion, all activities were within one standard deviation of EE. In the work of Nightingale and co-workers the EE for the propulsion at different wheelchair speeds (on a treadmill) was reported (42). Although the data cannot be compared directly to the values of our study, due to different protocols, we can see a linear relationship between wheelchair speed and EE in both studies, when excluding the task of wheeling at a speed of 6.5 km/h in our study. In conclusion, the SCI MET recorded in this study matches the values reported in the literature well. Therefore, we consider it a valid approach to use the measured EE of the present study for healthy lifestyle recommendations in the SCI population.

The most commonly used recommendation for an active lifestyle in the able-bodied population is the ACSM recommendation suggesting 30min of moderate to vigorous activity per day on at least 5 days per week. For the wheelchair-bound SCI population, the results of our study found wheeling at 3.5 or 5 km/h to represent an activity of moderate intensity. In order to choose one of the two speeds for the translation of the ACSM recommendation, we further examined the EE at these two wheeling speeds. According to Wilson et al. (56), 60 min of moderate to vigorous activity per day should result in ~150 kcal of increased EE, and 30 min of moderate to vigorous physical activity therefore corresponds to an additional 75 kcal. Wheeling at 5 km/h for 30 min was closer to the desired 75kcal than wheeling at 3.5 km/h. For this reason, we chose to use 5 km/h for the translation of the ACSM guidelines to the wheelchair-bound SCI population. This translation would therefore result in a recommendation to travel a daily distance of ~3 km at 5 km/h in the wheelchair.

There exist, however, also other recommendations for the able-bodied population. For the translation of the 10,000 steps/day (roughly 300 kcal/day) to a distance to travel per day in the wheelchair, we selected an average wheeling speed of 2 km/h. This value was based on daily averages obtained from long-term recordings in the SCI population (57, 58). Based on these values, the recommendation for the SCI population would be to wheel for around 8 km per day at this average speed. However, it is likely that this reference distance is far too high. First, the threshold of 10,000 steps/day is controversial in the able-bodied population, and some researchers have suggested lower thresholds for the able-bodied population. Second, the daily EE and the REE of an individual with SCI are lower than those of an able-bodied person, and therefore less than the additional 300 kcal/day (estimated for 10,000 steps) might be sufficient for a health-promoting effect. We further translated other activity recommendations for the able-bodied population such as an additional 150 and 200 kcal per day spent in activities of moderate to vigorous intensity, into daily distance to travel in the wheelchair. As these recommendations are, however, not well-established, we therefore did not consider them for our final recommendation.

Therefore, based on data of the present study we recommend to travel for at least 3 km at 5 km/h on 5 days a week in order to achieve a health-promoting additional daily EE. This recommendation is in line with the recommendations of the U.S. Department of Health and Human Services and the ACSM (53, 54). There are, however, wheelchair users who cannot achieve a wheeling speed of 5 km/h and therefore cannot fulfill the proposed combination of extra calories and intensity. Nevertheless, these wheelchair users could try to reach the goal of 3 km/day, first, because even at lower speeds they reach the recommended daily goal of additional 75 kcal, and second, because 3 km per day is more than what an average wheelchair user travels per day (around 2 km) (57, 58, 67, 68).

While our recommendation aims at minimizing the risk for cardiovascular diseases, Blair and co-workers stated in their work that 60 min instead of 30 min of moderate to vigorous physical activity per day is beneficial for different health outcomes such as for example, maintaining a lean body mass or improving muscular strength and endurance (55). In order to fulfill the 60 min recommendation, our results can easily be extrapolated to a daily distance to travel of 6 km/day.

We would like to acknowledge some limitations of this study. Firstly, the number of women included in this study (i.e., 3) may be considered too small (although it reflects a typical distribution in traumatic SCI) and, therefore, the sample measured may not be representative for the entire SCI population. Secondly, no individual HR calibration such as the method presented by Spurr and coworkers was used, which might have influenced the models including the HR (69). Thirdly, apart from the wheelchair wheel diameter, no information about the wheelchair types used by the study participants was included in the models, which might have influenced the EE estimation accuracy. Fourthly, the proposed algorithm does not consider the lifestyle of the individual (type and intensity of activities), which could have potentially improved our estimation model. The classification into different activity classes worked well for the activities included in this study, but a validation in a real-world setting is required. A further limitation of this study concerns the recommendations for a healthy lifestyle made for the SCI population. All values were translated from recommendations from the able-bodied population and it first has to be shown that those values should also be used in the SCI population. Finally, the REE models were based on 30 subjects only (equal to 30 data points), thus the inclusion of more subjects is needed to allow for the design of a more general estimation model.

## Conclusion

The models presented in this study accurately estimate EE in an unprecedented pool of 24 activities and in 3 h of continuous measurements in wheelchair-bound SCI individuals, making it a powerful tool to be used during continuous and non-obstructive recordings in real-world situations. IMU-based EE estimation is a promising methodology that may be used, together with the proposed wheeling reference value of 3 km per day, to promote a healthy lifestyle in SCI individuals at later stages of and/or after rehabilitation. The use of such recordings and recommendations may help to increase physical activity of SCI individuals to an extent allowing to decrease the prevalence of cardiovascular disease and increase quality of life in the long run.

## Author contributions

WP, MB, BW, CS, AC, MS, and RG designed the study, WP, LR, and MB collected the experimental data, WP and LR performed the data analysis, WP, LR, MB, BW, CS, AC, MS, and RG interpreted the results, revised the manuscript, and approved the final version.

### Conflict of interest statement

The authors declare that the research was conducted in the absence of any commercial or financial relationships that could be construed as a potential conflict of interest. The reviewer HS and handling Editor declared their shared affiliation.
